# Attributes of national governance for an effective response to public health emergencies: Lessons from the response to the COVID-19 pandemic

**DOI:** 10.7189/jogh.12.05021

**Published:** 2022-07-06

**Authors:** Yibeltal Assefa, Solomon Woldeyohannes, Katherine Cullerton, Charles F Gilks, Simon Reid, Wim Van Damme

**Affiliations:** 1 School of Public Health, the University of Queensland, Brisbane, Australia; 2 Institute of Tropical Medicine, Antwerp, Belgium

## Abstract

**Background:**

The Coronavirus Disease 2019 (COVID-19) pandemic takes variable shapes and forms in different regions and countries. This variability is explained by several factors, including the governance of the epidemic. We aimed to identify the key attributes of governance in response to the COVID-19 pandemic and gain lessons for an effective response to public health emergencies.

**Methods:**

We employed a mixed-methods design. We mapped the attributes of governance from well-established governance frameworks. A negative binomial regression was conducted to identify the effect of the established governance measures on the epidemiology of the COVID-19 pandemic. We used publicly available data on COVID-19 cases and deaths in countries around the world. Document review was conducted to identify the key approaches and attributes of governance during the pre-vaccine era of the response to the COVID-19 pandemic. We conducted a thematic analysis to identify key attributes for effective governance.

**Results:**

The established governance measures, including generation of intelligence, strategic direction, regulation, partnership, accountability, transparency, rule of law, control of corruption, responsiveness, effectiveness, efficiency, equity, ethics, and inclusiveness, are necessary but not sufficient to effectively respond to and contain the COVID-19 pandemic. Additional attributes of national governance were identified: 1) agile, adaptive, and transformative governance; 2) collective (collaborative, inclusive, cooperative, accountable, and transparent) governance; 3) multi-level governance; 4) smart and ethical governance: sensible, pragmatic, evidence-based, political, learner, and ethical.

**Conclusions:**

The current governance frameworks and their attributes are not adequate to contain the COVID-19 pandemic. We argue that countries need agile, adaptable, and transformational, collaborative, multi-level, smart and ethical governance to effectively respond to emerging and re-emerging public health threats. In addition, an effective response to public health emergencies depends not only on national governance but also on global governance. Hence, global health governance should be urgently renewed through a paradigm shift towards universal health coverage and health security to all populations and in all countries. This requires enhanced and consistent global health diplomacy based on knowledge, solidarity, and negotiation.

The Coronavirus Disease 2019 (COVID-19) pandemic demonstrates that the world remains vulnerable to public health emergencies with significant health and other socio-economic impacts. The COVID-19 pandemic takes variable shapes and forms in how it affects communities in different regions and countries [1,2]. As of September 15, 2021, over 226 million cases and over 4.6 million deaths were reported since the start of the pandemic. The number of cases per million population ranged from 8965 in Oceania to 85 712 in South America while the number of deaths per million population ranged from 116 in Oceania to 2627 in South America. The number of cases also ranged from one case in Micronesia to more than 42 million cases in the United States [3].

The variable expression of the pandemic across countries is explained by several factors, including governance and leadership of the epidemic response, as well as epidemiological, demographic, health system, and socio-economic factors [4]. Ending the pandemic in a country necessitates not only good prevention and treatment measures, but also effective and targeted public health emergency governance [5]. The pandemic has challenged the current systems of both global and national governance. These challenges speak of the crisis of leadership and the limitations of contemporary democracies [6]. An independent panel report indicated that the global response to COVID-19 was too slow and that leadership was deficient. The report highlighted the issues with global governance of COVID-19, namely: the World Health Organization’s position, structure, and lack of financing; a lack of coordinated and sufficient financing for pandemic preparedness and response; inequities; and limitations in the broader global health architecture [7].

Governance encompasses the relationships between government and society, including the means through which private actors, markets, and interest-based networks influence policy decisions [8]. Governance is identified as a crucial factor for explaining the heterogeneity of the COVID-19 pandemic among countries with high human development index (HDI) [9]. In these countries, there has been substantial variation in the nature and timing of the public health responses implemented [10]. Countries with the poorest results, such as the United States and Brazil, had uncoordinated approaches that devalued science and denied the potential impact of the pandemic [11,12]. Only a minority of countries, such as Australia and South Korea, have implemented coordinated measures and managed to contain and stop the spread of the virus [13].

In countries which demonstrated a more effective epidemic response, governance quality not only influenced how they responded but also had a moderate impact on how well their policies were implemented [14]. Furthermore, it was found that countries with better governance continue to be more resilient during the COVID-19 crisis [15]. Overall, countries that have fared the best are the ones with good governance and public support [16]. Moving forward, it is important that countries learn from their epidemic response and from each other. However, there is no currently uniform understanding of the approaches and attributes of governance for an effective response to public health emergencies. In this paper, we aimed to understand the key attributes of national governance for an effective response to COVID-19 and other public health emergencies.

## METHODS

We employed a mixed-methods (including quantitative and qualitative approaches) design to 1) determine the association between COVID-19 and governance and 2) identify the key attributes of governance for an effective COVID-19 response. We understand that the response to the pandemic has two phases:1) before vaccines existed and 2) since vaccines have become part of the response. The study has focused on the first phase of the pandemic where there were no vaccines.

The study was conducted in three stages. We first mapped the attributes of governance in existing frameworks. We then conducted a regression analysis to assess the association between these existing attributes of governance and COVID-19 cases and deaths. Finally, we conducted a review of the literature to identify key attributes of governance in countries with lower cases and deaths due to COVID-19.

### Mapping of governance attributes against existing frameworks

We mapped the attributes of well-established governance parameters employed by the World Health Organization (WHO) [17], the World Bank [18], and the United Nations Development Program (UNDP) (**Table 1**) [19]. This will be the basis for the subsequent quantitative analysis and document review. According to these institutions, governance refers to the exercise of political and administrative authority, comprising the mechanisms, processes and institutions, to articulate interests, exercise legal rights, meet obligations, and mediate differences [19]. Attribute mapping was conducted by YA and SW to identify the indicators for governance. YA and SW agreed to include all attributes of governance used by WHO, the World Bank, and UNDP.

**Table 1:** Mapping of attributes of governance

Global Institutions	Attributes of governance
**World Health Organization**	➢ Generation of intelligence
	➢ Formulating strategic policy direction
	➢ Ensuring tools for implementation:
	• Powers
	• incentives
	• sanctions
	➢ Building coalition/building partnership
	➢ Ensuring a fit between policy objectives and organizational structure and culture
	➢ Ensuring accountability
**World Bank**	➢ Process by which those in authority are selected and replaced:
	• Voice and accountability
	• Political instability and violence
	➢ Ability of the government to formulate and implement sound policies:
	• Government effectiveness
	• Regulatory burden
	➢ Respect of citizens and the state for institutions which govern their interaction:
	• Rule of law
	• Graft (control of corruption)
United Nations Development Program	➢ Legitimacy and voice:
	• Participation
	• Consensus orientation
	➢ Direction:
	• Strategic vision
	➢ Performance:
	• Responsiveness
	• Effectiveness and efficiency
	➢ Accountability:
	• Accountability (decision-makers in government, the private
	• sector and civil society organizations)
	• Transparency
	➢ Fairness:
	• Equity and inclusiveness
	• Rule of law

### Quantitative analysis

We conducted a regression analysis to identify the role of the established attributes of governance in the COVID-19 response. COVID-19 cases and deaths were the dependent variables while governance attributes and their indices recommended by the World Bank, which has a more widely utilized governance index than WHO and UNDP, were the primary independent variables. The WHO and UNDP frameworks for governance do not have indices yet. We included key variables, such as universal health coverage index, global health security index, human development index, prevalence of obesity, proportion of the population living in urban areas, and percentage of elderly population, in the models to adjust for confounding due to these variables.

We fitted both Poisson and negative binomial regression models to model the COVID-19 cases and deaths. Over-dispersion, which means the conditional variance exceeds the conditional mean (dispersion parameter greater than one for both linear and quadratic transformation approaches) [20], was found for the COVID-19 cases and deaths outcomes; hence, we presented the results from the negative binomial regressions. A linear regression model was fitted to model the COVID-19 case fatality rate (CFR) for countries around the world.

We used the publicly available secondary data sources from Johns Hopkins University (https://coronavirus.jhu.edu/data/new-cases) for COVID-19 and UNDP 2020 HDI report (http://hdr.undp.org/en/2019-report) for HDI, demographic and epidemiologic variables. These data sources are regularly updated, open-source, and utilized by researchers, policymakers, and funders. We used the World Bank’s database (http://info.worldbank.org/governance/wgi/Home/Reports) for specific and overall indices for attributes of governance.

### Document review

We conducted a narrative review of the literature on COVID-19 governance to identify key attributes of governance and leadership that have played a critical role in the epidemic response in countries which have had relatively lower cases and deaths. We used the medians of cases and deaths to determine countries with relatively higher or lower cases and deaths of COVID-19. We conducted the search using the following search terms: COVID-19 and governance. The search was conducted in the following databases: PUBMED, Scopus, and EMBASE. YA and SW executed the review. We then conducted a thematic analysis of whole papers to identify the attributes of governance for an effective response to the COVID-19 epidemic. Codes and themes were identified and discussed between the two reviewers. We also checked for the presence of these attributes in countries with relatively higher COVID-19 cases and death.

## RESULTS

The findings of the study are organized in three parts: 1) mapping of governance attributes; 2) regression analysis; and 3) document review.

### Mapping of established governance attributes

**Table 1** summarises the established governance attributes recommended by the World Health Organization, the World Bank, and the United Nations Development Program. We have identified that the attributes from the global institutions are similar in content though they may use different terms.

### Regression analysis

The negative binomial regression indicates that the established governance measures do not have a significant effect (incidence rate ratio (IRR)) on COVID-19 cases and deaths even though they have negative effects after adjusting for other non-governance variables (**Tables 2** and **3**). The linear regression also indicates that these established governance measures do not have a statistically significant effect on case-fatality rate (CFR) even though they have negative effects after adjusting for other non-governance variables (**Table 4**).

**Table 2:** Negative Binomial Regression for total COVID-19 cases

						95% CI	
	**Estimate**	**Std. Error**	**z value**	***P*-value**	**IRR**	**LCL**	**UCL**
(Intercept)	-10.424	0.918	-11.358	0	0	0	0
Voice and Accountability	0.27	0.143	1.893	0.058	1.31	0.99	1.734
Political Stability	-0.203	0.149	-1.359	0.174	0.816	0.609	1.094
Government Effectiveness	0	0.331	0.001	0.999	1	0.523	1.913
Rule of Law	0.109	0.339	0.321	0.748	1.115	0.573	2.169
Control of Corruption	-0.33	0.27	-1.221	0.222	0.719	0.423	1.221

Std. Error – standard error, IRR – incidence risk/rate ratio, LCL – lower confidence limit, UCL – upper confidence limit

**Table 3:** Negative Binomial Regression for total COVID-19 deaths

						95% CI	
	**Estimate**	**Std. Error**	**z value**	***P*-value**	**IRR**	**LCL**	**UCL**
(Intercept)	-14.309	0.922	-15.526	0	0	0	0
Voice and Accountability	0.574	0.146	3.934	0	1.776	1.334	2.365
Political Stability	-0.206	0.155	-1.324	0.186	0.814	0.6	1.104
Government Effectiveness	-0.521	0.336	-1.548	0.122	0.594	0.307	1.149
Rule of Law	0.292	0.351	0.832	0.405	1.339	0.673	2.662
Control of Corruption	-0.593	0.276	-2.146	0.032	0.553	0.322	0.95

Std. Error – standard error, IRR – incidence risk/rate ratio, LCL – lower confidence limit, UCL – upper confidence limit

**Table 4:** Linear Regression for Case Fatality Rate

	Estimate	Std. Error	t value	*P*-value
(Intercept)	-2.194	2.296	-0.956	0.341
Voice and Accountability	-0.397	0.291	-1.364	0.175
Political Stability	-0.095	0.323	-0.295	0.768
Government Effectiveness	-0.437	0.671	-0.652	0.515
Rule of Law	-0.983	0.857	-1.147	0.253
Control of Corruption	0.301	0.772	0.39	0.697

Std. Error – standard error

### Document review

A total of 45 papers analysing national governance of COVID-19 were included in this review. The review has identified the following national governance attributes in countries with a relatively lower COVID-19 cases and associated deaths: 1) agile, adaptive, and transformative governance and leadership; 2) collective governance and leadership: collaborative, inclusive, cooperative, accountable, and transparent governance; 3) multi-level governance: decentralized; 4) smart leadership and governance: sensible, pragmatic, evidence-based, political will, learner, and ethical (**Table 5**).

**Table 5:** Attributes and characteristics of effective governance in COVID-19

Attributes of Governance	Characteristics of Governance
Agile, adaptive, and transformative	• Agile
	• Adaptive
	• Transformative
Collectivism (whole-of-government and whole-of-society)	• Collaborative
	• Inclusive
	• Cooperative
	• Transparent
	• Accountable
Multi-level governance	• Decentralized:
	o Health facility
	o Community-based
	• Accountable
Smart- and ethical-governance	• Sensible, cognizant and pragmatic
	• Science-based and rapid innovations
	• Political
	• Ethical:
	o equity
	o solidarity
	o subsidiarity
	o stewardship
	• Learning from past pandemics

#### Agile, adaptive, and transformative governance

The surge of COVID-19 cases in some countries with excellent health infrastructure suggests that a successful response should go beyond strengthening the health infrastructure [21]. Countries have had to respond to the COVID-19 outbreak with limited information and many uncertainties. Agile and adaptive governments seem better able to respond to their epidemics [22,23]. The South Korean government employed an agile and adaptive approach to mitigate the surge of COVID-19 [24].

We have identified that adaptive leadership and pivots are essential when considerable uncertainty in the appropriate design and implementation of health services is present [25]. Transformative and experimental forms of governance emerged in Switzerland, when administrative routines and established management techniques dissolved during the COVID-19 epidemic [26]. The coronavirus pandemic has transformed Australia’s health system as new ways of working were implemented with unbelievable speed. The upgrading of the meeting between the Prime Minister and state and territory leaders – from the Council of Australian Governments to a ‘national cabinet’ meeting at least once a week – was critical in the transformation [27].

On the other hand, there were limitations to governance arrangement in India’s COVID-19 crisis. Adaptive governance, delivered through a multidimensional and integrated health system agenda setting, was necessary to overcome the challenges of the country’s epidemic [28]. In Bangladesh, an adaptive response strategy allowed old and new networks of organizations to align and work collectively [29].

#### Collective governance and leadership: collaborative, inclusive, cooperative, transparent

COVID-19 has demonstrated the weaknesses of extreme individualism in fighting a pandemic, which requires a coordinated and unified public response. East Asia has been a global leader in preventing the spread of COVID-19 because of a vigilant public compliant with public safety measures [30]. African Union leaders also coordinated their responses, and developed a continent-wide African Medical Supplies Platform [31].

China is one of the countries that successfully contained the virus, with a regained economic vitality, through strong leadership, clear accountability, swift social mobilization, high levels of public trust, and effective communication [32]. The response has been guided by a whole-of-government response with a multi-sectoral cooperation platform, and whole-of-society actions with engagement of community organizations and citizens [33]. Collective action has been facilitated through moral obligation (through internalizing epidemic prevention and control norms) and public leadership (through professional training and by motivating village elites) [34].

South Korea had greater success in controlling the epidemic compared to other countries due to its collaborative governance approach that brought synergy across the entire governance system, including both public and private ones [35]. For swift and effective management of the pandemic, the South Korean government utilized partnerships with the private sector [36], with the leadership rapidly mobilizing public and private means for survival [37]. The South Korean government also responded to COVID-19 with transparency in communicating risk and citizens' voluntary cooperation, which are critical factors to mitigate the surge of COVID-19 [24]. Collaborative governance has enabled Taiwan to fight against COVID-19, which resulted in relatively low infection and death numbers. Taiwan streamlined a Command Centre in a timely manner to launch its response, mobilize the public, and engage private resources to implement the strategies and policies, which were further enhanced by collaborative behaviours and volunteers [38].

#### Multi-level governance is key in COVID-19 pandemic response

The response to a public health crisis, which is characterized by systematic, cross-border and uncertain actions, response and outcomes, creates tension between centralization of power and decentralization of governance for an effective response [39]. COVID-19 has forced health systems (including local health systems, hospitals, and primary health care) to move towards decentralization [40]. China has adopted grid governance measures, which can link management resources from top to bottom and be implemented quickly in urban and rural communities. China's local governments are combating COVID-19 with unprecedented responses. This measure helped China bring the pandemic under control within its own borders; and, as a result, residents' lives and factory production gradually began to return to normal [41]. Wenzhou state is a very good example for taking timely, rigorous, and systematic measures in fighting the spread of this epidemic within its jurisdiction [42].

On the other hand, the COVID-19 crisis in India is partly explained by the limitations of the centralised governance arrangement, which cannot account for the complexity of the health system. It was identified that the pandemic response necessitated increased operational decentralisation, which needs to be reinforced with greater autonomy, consultation, cooperation and coordination among different level actors [28]. The pandemic has also illustrated the need for liberating local clinical leaders and managers and empowering them to find their own solutions [43]. Both administration and frontline personnel are necessary for successful management of the COVID-19 pandemic [44,45]. Local governments have galvanized new kinds of interaction and coordination between administrative units involved in the implementation of disease control and social security mechanisms [46]. Effective multi-level communication is valuable for conveying new policies, training, and workflows [47].

#### Smart- and ethical-governance: Sensible, evidence-based, political, learner, and ethical

We have also identified that smart leadership, as well as fragmentation, segmentation and insufficiency of health systems, explains the variability in the epidemiology of COVID-19 around the world [48]. Smart leadership encompasses: making sense of a crisis, evidence-based leadership, political will, learning from past pandemics, and ethics (solidarity, subsidiarity, and stewardship). These are presented one-by-one below.

***Making sense of a crisis:*** Although the virus is a ubiquitous problem, world leaders have varied appreciably in their responses. A leader's sensemaking approach has influenced responses to COVID-19, and is a critical element in successful crisis management efforts [49]. The pandemic has shown the devastating consequences both of a lack of public health preparedness and incompetent leadership. Public health “blind spotting” has emerged as a significant threat to population health. Lack of awareness of or ignoring crises will cause long-lasting harm. An effective response to crises requires competent political leadership, public health cognizance and evidence-based strategies, based not only on public health data but also on the education of leaders at all levels of society [50]. Decision-making processes using a diversity of knowledge can help with making quick decisions and improve trust, satisfaction, and engagement [51]. The Australian government was aware that ‘business as usual’ would not be enough to adequately respond to the pandemic; without dramatic action, hospitals would have been overwhelmed. This awareness has transformed Australia’s response to contain the epidemic as new ways of working were implemented with implausible momentum [27].

***Leadership, science, and political will:*** Bold public health leadership, advances in science and rapid innovations, as well as courageous political will are needed to end the COVID-19 pandemic [52]. While politics and science often conflict, effective leadership should be supported and sustained in the best interests of health officials and, even more importantly, the communities they serve. There is no better time than now to unite the polarities of science and politics. We cannot easily end the pandemic if we do not follow science and allow scientific principles to guide and inform the path forward [53]. The pandemic has highlighted the need for leaders to be educated on implementation science principles so that they are able to make evidence-based decisions [54]. Compared to many other countries, Norway performs well in handling the crises thanks to its competent politicians, a high-trust society with a reliable and professional bureaucracy, a strong state, a good economic situation, a big welfare state, and low population density. The presence of these factors allowed a valuable balance between governance capacity and legitimacy [55].

***Sense of shared social identity*:** Leaders' ability to bring the pandemic under control is grounded in their ability to create and embed a sense of shared social identity and represent and advance the shared interests of society. Leaders need to recognize the importance of shared identity, then build and sustain this through their actions, provide support to the group and its members, prepare the group materially and psychologically for a crisis, and deliver outcomes that matter for them [56]. Effective leadership in the Arab ethnic minority in Israel has reduced this group's vulnerability and built resilience. The Arab ethnic minority makes up 21% of Israel's population yet comprised just 8.8% of confirmed cases and 3.6% of deaths from COVID-19, despite their higher risk profile. This contrasts with reports of increased mortality in ethnic minorities and economically disadvantaged populations in other countries. This is facilitated through effective leadership and cooperation between individuals and institutions, particularly engagement of community and religious leaders [57].

***Ethical leadership***: Values and ethical behaviours, including solidarity, subsidiarity, and stewardship, are vital in adequately and sustainably containing the pandemic. There are widespread reports of disproportionate impacts of the COVID-19 pandemic among already vulnerable communities worldwide. Disadvantaged people are at higher risk of infection and death from COVID-19, and they have less access to care due to systems that treat health as a commodity and not a human right [58]. Countries can learn from ethical health leadership missteps that occurred during COVID-19. Delivering quality health care to the population occurs when health inequalities are reduced. Health inequalities driven by the lack of good governance including corruption, incompetence, and mismanagement of resources were persistent during the COVID-19 pandemic. However, ethical health leadership does not occur in a vacuum; it requires concerted ethical economic and political leadership [59]. Healthcare delivery happens within complex settings which are riddled with systemic political and economic challenges [59]. Solidarity, subsidiarity, and stewardship should be guiding principles and ethical foundations in times of pandemics [60]. These principles have been observed in several low-income countries, such as Rwanda, which managed to take significant steps to limit the spread of the virus, rolled out a complete nationwide lockdown, while also providing social support to vulnerable populations [54].

***Learning from past pandemics:*** South Korea’s response has benefited from its lessons in previous pandemics. The lessons have influenced the country to have both proactive and reactive strategies [61]. For swift and effective pandemic management, the South Korean government utilized partnerships with the private sector [36]. This contributes to turning a pandemic crisis into an opportunity for sustainable leadership [37] (**Table 5**).

On the other hand, countries with an absence of governance with the above attributes, including coherent leadership and social trust, have worse outcomes [62]. Brazil's governance of the COVID-19 epidemic has been described as disastrous. President Bolsonaro's dangerous science denialism has plunged the country into catastrophe [63]. The erosion of trust in the United States government has hurt the country’s ability to respond to the COVID-19 crisis. President Donald Trump ran the risk of eliminating important administrative expertise which could help manage the crisis adequately. This left the administration far less prepared to address the COVID-19 epidemic than it otherwise could have been with these experts [64,65].

## DISCUSSION

This study aimed to understand the attributes of governance in an effective response to COVID-19. It identified that effective governance has more attributes than those recommended by WHO, the World Bank, and the United Nations Development Program. The current governance attributes recommended by these global institutions include generation of intelligence, strategic direction, intelligence, regulation, partnership, accountability, transparency, rule of law, control of corruption, responsiveness, effectiveness, efficiency, equity, ethics, and inclusiveness. These governance frameworks and their attributes are primarily designed to deal with routine and ordinary situations. They are essential, but not sufficient to effectively manage extraordinary situations, such as public health emergencies. Additional governance attributes are identified to be vital for an effective response to COVID-19. We thus propose an expanded framework for the governance of public health emergencies, with the following attributes to supplement the already existing attributes recommended by the global health institutions: agile, adaptable, transformational, collaborative, multi-level, smart, and ethical governance. These attributes are essential to end the current pandemic and to prepare adequately to prevent, detect and respond to emerging and re-emerging public health threats [66].

The proposed expanded framework implies that it is vital to include agility, adaptability, and transformation in public health emergencies governance. Agility relates mainly to the speed of response within given structures. Adaptive leadership is relevant when there is considerable uncertainty in the design and implementation of health services [25]. Adaptive leadership is an iterative process, taking a wide view of the situation, interpreting data from multiple directions, and taking real-time action. Adaptive governance calls for both decision speed and sound analysis, for both centralized and decentralized decision-making, for both innovation and bureaucracy, and both science and politics [67]. For instance, the South Korean government employed an agile and adaptive approach to mitigate the surge of its COVID-19 epidemic [24]. Transformational governance is defined as the capacity and capability to develop initiatives that can keep up with continuously changing social contexts [68]. This is achieved through increased risk tolerance, significant systemic investment, and restructured economies and power relations. Switzerland dissolved its administrative routines and established transformative and experimental forms of governance [26], while Australia transformed its response by upgrading the Council of Australian Governments to a ‘national cabinet’ [27]. These attributes of governance are possible if the ‘why’ question is clearly answered and felt by everyone involved in the response [69]. The leadership should also take advantage of new technologies for an effective pandemic response while, at the same time, taking precautions towards an equitable and ethical use of technology [70].

Governance of the COVID-19 response calls for reorientation towards more collaborative, inclusive and participatory engagement [71]. It is vital that the leadership strengthens a comprehensive capacity, including diseases control expertise and political skills, to anticipate significant disruptions, provide a clear perspective on what is happening and what response is needed, bring stakeholders together and mobilize the population to act effectively. The leadership must recognise the multidimensional effects and needs of the society during crises and consult with a broader public by listening to the voices of populations at risk of getting left behind due to structural and other factors [72]. An inclusive and transparent leadership is essential in reducing psychological distress [73]. The response requires a balance between centralized coordination (with a command centre) and decentralized responses. A strategic decision to mobilize the private sector to help with the response can also be beneficial, with an appropriate regulatory framework. All this should be facilitated by frequent communication that builds trust and fuels engagement among actors while helping lessen the spread of misinformation and fear in the society [74,75].

The COVID-19 pandemic can be viewed as an unprecedented catalyst for social transformation that underscores the need for multi-level and cross-sectoral solutions to address health inequity due to systemic challenges [76]. These inequities must be changed through strategic, multi-level, multi-sectoral and interdisciplinary leadership, as well as community engagement, research and innovation [77]. Building stronger universal health coverage systems and policies is essential and should be coordinated with social protection systems. This requires system-wide policies, breaking the boundaries of traditionally fragmented welfare systems and health systems and programs [78].

We acknowledge that an effective response to pandemics requires arrangements and attributes of governance beyond national governance [79]. The COVID-19 pandemic demonstrates that an effective response to public health emergencies requires not only national and local but also global governance [79]. However, global health governance is at crossroads due to challenges of fragmentation, nationalism, and individualism [8,80]. Effective response to the pandemic demands global cooperation and solidarity through pooling and sharing resources [81]. Transparency in sharing relevant information among countries is essential [82]. The pandemic responses have highlighted the critical importance of data systems and digital capacity in coordinating the response [83]. Renewed global (health) governance mechanisms through political and legal solutions are urgently needed to truly encourage international collaboration [46]. It is vital to rethink and develop new national and international governance strategies [84], which can facilitate a international cooperation system reform [85,86]. In our deeply interwoven and interdependent world, the only way to defeat the COVID-19 pandemic and prepare for future pandemics is for all countries to work together in a spirit of solidarity and cooperation [42]. This is possible through global health diplomacy, which requires diplomatic and negotiation skills as well as public health knowledge [87]. Furthermore, the world needs a new framing for global health security based on “One Nature” and “One Health” governance approaches [88].

## CONCLUSIONS

Governance plays a critical role in the COVID-19 pandemic response; however, the current governance frameworks and measures are inadequate for understanding national governance of the COVID-19 epidemic. We argue that an expanded framework (including agile, adaptive, transformative, collaborative, multi-level, smart and ethical) is needed for an effective national governance of public health emergencies. However, an effective response to public health emergencies depends on not only national but also global governance. Hence, renewed global health governance mechanisms are urgently needed through a paradigm shift in the framing of global health towards universal health coverage and health security to all populations in every country. This requires enhanced global health diplomacy based on knowledge, solidarity, and negotiation.
